# Molecular Basis of the Schuurs–Hoeijmakers Syndrome: What We Know about the Gene and the PACS-1 Protein and Novel Therapeutic Approaches

**DOI:** 10.3390/ijms23179649

**Published:** 2022-08-25

**Authors:** María Arnedo, Ángela Ascaso, Ana Latorre-Pellicer, Cristina Lucia-Campos, Marta Gil-Salvador, Ariadna Ayerza-Casas, María Jesús Pablo, Paulino Gómez-Puertas, Feliciano J. Ramos, Gloria Bueno-Lozano, Juan Pié, Beatriz Puisac

**Affiliations:** 1 Unit of Clinical Genetics and Functional Genomics, Department of Pharmacology-Physiology, School of Medicine, University of Zaragoza, CIBERER-GCV02 and IIS-Aragon, E-50009 Zaragoza, Spain; 2 Unit of Paediatric Cardiology, Service of Paediatrics, Hospital Universitary Miguel Servet, E-50009 Zaragoza, Spain; 3 Molecular Modelling Group, Center of Molecular Biology “Severo Ochoa” (CSIC-UAM), Cantoblanco, E-28049 Madrid, Spain; 4 Department of Pediatrics, Hospital Clínico Universitario “Lozano Blesa”, E-50009 Zaragoza, Spain

**Keywords:** Schuurs–Hoeijmakers syndrome, SHMS, *PACS1*-NDD, trafficking protein, targeted therapy

## Abstract

The Schuurs–Hoeijmakers syndrome (SHMS) or *PACS1* Neurodevelopment Disorder (*PACS1*-NDD) is a rare autosomal dominant disease caused by mutations in the *PACS1* gene. To date, only 87 patients have been reported and, surprisingly, most of them carry the same variant (c.607C>T; p.R203W). The most relevant clinical features of the syndrome include neurodevelopment delay, seizures or a recognizable facial phenotype. Moreover, some of these characteristics overlap with other syndromes, such as the PACS2 or Wdr37 syndromes. The encoded protein phosphofurin acid cluster sorting 1 (PACS-1) is able to bind to different client proteins and direct them to their subcellular final locations. Therefore, although its main function is protein trafficking, it could perform other roles related to its client proteins. In patients with *PACS1*-NDD, a gain-of-function or a dominant negative mechanism for the mutated protein has been suggested. This, together with the fact that most of the patients carry the same genetic variant, makes it a good candidate for novel therapeutic approaches directed to decreasing the toxic effect of the mutated protein. Some of these strategies include the use of antisense oligonucleotides (ASOs) or targeting of its client proteins.

## 1. Introduction

The Schuurs–Hoeijmakers syndrome (SHMS), or *PACS1* Neurodevelopmental Disorder (*PACS1*-NDD) (MIM# 615009), is a rare autosomal dominant disease [1], which has recently been included in a group of genetic disorders of cellular trafficking [2]. It was firstly described in 2012 in two patients with intellectual disability (ID) and a striking facial resemblance; they both carried the same mutation in the *PACS1* gene [1]. To date, less than a hundred patients with *PACS1*-NDD have been genetically diagnosed, and, surprisingly, only three pathogenic variants have been reported [1,3,4,5,6]. Furthermore, most of the patients have been described in the last few years, when the *PACS1* gene was included in the gene panel analysis for neurodevelopmental disorders; therefore, it is possible that the prevalence of this syndrome could be underestimated.

Although the clinical characteristics of *PACS1*-NDD patients are well described, there is little evidence on the pathomolecular mechanisms of this disease. The affected protein, phosphofurin acid cluster sorting 1 (PACS-1), belongs to a family of two members of membrane and protein trafficking regulators [7]. It was initially discovered in 1998 [8], and it has been reported in metazoans, invertebrates [9] and vertebrates [10]. Lower species possess only a single gene of the *PACS* family; however, the *PACS* gene was duplicated with the appearance of vertebrates, resulting in the *PACS1* and *PACS2* genes [10].

PACS-1 is a multifunctional membrane traffic regulator that plays an important role in cellular homeostasis [8,10]. An initial function of PACS-1 was the transport of several proteins between endosomes and the trans-Golgi network (TGN) [7]. However, in the last few years, novel roles have been proposed, such as a Ca^2+^ flux regulator or its probable implication in genomic stability [10,11,12]. The knowledge of the molecular mechanisms implied in the development of the *PACS1*-NDD is essential to make proper phenotype–genotype correlations and to propose therapies that could help and improve the quality of life of these patients. In this review, we will carry out a detailed study of the functions already known of the PACS-1 protein, of its role in the progression of the disorder and the latest improvements in the development of successful therapeutic strategies.

## 2. Clinical Characteristics of *PACS1*-NDD

The *PACS1*-NDD was first described in two unrelated male patients in 2012 [1]. Since then, about 100 patients have been reported in the literature [1,3,4,5,6]. All patients are described have a neurodevelopmental delay with an intellectual disability and psychomotor retardation. The range varies from mild to severe, although most of them have a moderate delay [13]. Language skills are universally affected, more severely than motor skills. Hypotonia is reported in approximately one third of the subjects, and it improves over time [13]. However, one patient has been described as having an impairment in their walking abilities over time [3]. Individuals with the *PACS1* mutation interact with others and appreciate receiving personal affection, but also show behavioral difficulties. Seizures are a common clinical feature, affecting 60 percent of patients; most of them are generalized as tonic-clonic, and are well controlled with antiepileptic drugs. Brain abnormalities have been found, mostly cerebellar. Other findings include ventriculomegaly, hydrocephalus or atrophy of the corpus callosum [3,5,14,15,16,17,18].

In addition to the neurological disorders, patients show a characteristic facial phenotype, which is easily recognizable by a clinician. It is characterized by full and arched eyebrows, hypertelorims with downslanting palpebral fissures, long eyelashes, a bulbous nose, a flat philtrum and large low-set ears [13].

Patients also suffer from other congenital anomalies, from cardiac anomalies to ocular alterations, where septal defects and coloboma stand out. They may also have skeletal abnormalities (abnormal skull shape), cryptorchidism or feeding problems, among others [3,19]. These clinic characteristics are summarized in Table 1.

The strong similarity between *PACS1*-NDD, PACS2 syndrome and the recently diagnosed Wdr37 syndrome is striking. The three disorders share a very similar facial gestalt, intellectual disability, neurodevelopmental delay and seizures, suggesting that they might be included in the same disease spectrum [20,21,22,23,24,25]. Moreover, a new group of diseases, caused by cellular trafficking defects and characterized by neurodevelopmental disorders and skeletal abnormalities, has been recently proposed [2,20,21,22,23,24,25,26]. On the other hand, there are other closely related clinical syndromes, including Cornelia de Lange, Kabuki or Coffin-Siris, although they do not share the facial gestalt (Table 1).

## 3. Molecular Basis of the Disease 

### 3.1. Genetic Update

To date, 87 patients with a *PACS1* deficiency have been genetically diagnosed, and, surprisingly, most of them carry the same pathogenic variant, the missense c.607C>T (p.R203W). This variant has been clearly demonstrated to be pathogenic because none of the parents tested were carriers [3], which means that all patients had a de novo mutation. There is one other pathogenic variant, c.608G>A, reported in only one patient, which results in a change in the same position, but the amino acid change is different, p.R203Q [5]. These patients have most of the typical features of the *PACS1*-NDD (Table 1). Lately, two novel missense variants have been reported, one in the ClinVar database, the c.1574G>A (p.R525K), whose relationship with the *PACS1*-NDD has to be studied [27]. The other is in a broad study about the autism spectrum disorder (p.R245W) without a specific phenotype of the disease [28].

More recently, a multi-exon deletion of *PACS1* has been reported [6]. Liu and Cols found in four members of a three-generation pedigree the deletion of the exons 12 to 24 in the *PACS1* gene. However, the phenotype of this variant was milder, with slight speech and cognitive delay, only affecting two members of the last studied generation [6]. Furthermore, several databases have reported chromosomal reorganizations where the *PACS1* gene is involved (ClinVar, DECIPHER) [27,29]. Nevertheless, the huge number of affected genes does not allow us to directly relate the patients’ phenotype with the *PACS1* gene.

### 3.2. PACS1 Gene Regulation

*PACS1* is a gene that is broadly expressed in human tissues (GTEx database) [30]. According to the BrainSpan and EvoDevo databases, its mRNA expression is upregulated during fetal brain and cerebellum development, and it decreases after birth to slightly increase in puberty [31,32]. Its expression level is also important in pubertal testis tissues [31,32]. This specific tissue distribution could be the reason for some of the clinical characteristics of *PACS1*-NDD patients.

The *PACS1* gene is located on 11q13.1 and contains 24 exons and at least 16 transcripts (ensembl) [33]. Its expression regulation has been poorly studied, but some proteins have been proposed as regulators of PACS-1 expression, such as the P300/CBP-associated factor (PCAF) or the transcriptional adaptor protein 3 (ADA3). Chromatin inmuno precipitation (ChIP) experiments showed an enrichment in the promoter of *PACS1* of the proteins PCAF and ADA3. The downregulation of both factors decreased the relative *PACS1* expression level in HeLa and HCT116 cell lines. These facts point to a plausible role of PCAF and ADA3 as gene regulators of *PACS1* [34].

In addition to that, the study of its 3′-UTR sequence showed two putative binding sites for the miRNAs, 34a and 449a. In tumor tissues, an overexpression of these miRNAs and a downexpression of the PACS-1 protein has been reported [35]. Furthermore, the overexpression of another miRNA, miR-485-5p, induces a decrease of PACS-1 in pericytes that has been related to Alzheimer’s disease progression [36].

### 3.3. Characteristics of the PACS-1 Protein

**Figure 1:**
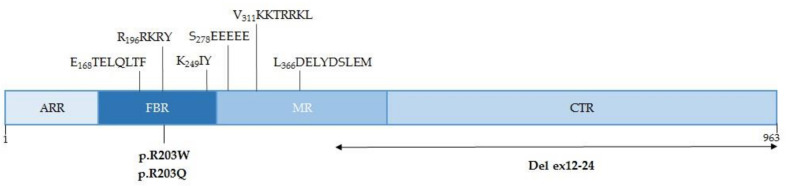
Schematic representation of the PACS-1 protein. Key sequences and pathological variants are located in the sequence. ARR, atrophin-1-related region; FBR, furin-binding region; MR, middle region; CTR, *C*-terminal region.

PACS-1 is a protein of 963 amino acids with several domains and key regions located in the cytosol and nucleus [12,37]. Its N-terminal region, called ARR (atrophin-1-related region), is followed by the FBR (furin-binding), the MR (middle) and the CTR (C-terminal) domains (Figure 1). The FBR binds client proteins, such as furin, as well as the cytoplasmic membrane trafficking machinery. Several specific sequences have been described, which are responsible for the binding of PACS-1 to the clathrin adaptors AP-1 and AP-3 (E_168_TELQLTF) or to the monomeric adaptor GGA3 (K_249_IY). Moreover, in the FBR is the binding sequence of the protein kinase CK2 (R_196_RKRY) that phosphorylates the S_278_ residue located in the MR autoregulatory domain (S_278_EEEEE). Furthermore, the MR also has a nuclear localization sequence (NLS) (V_311_KKTRRKL) and a nuclear export sequence (NES) (L_366_DELYDSLEM) [37]. The entry and exit from the nucleus of PACS-1 depends on the receptors importin alpha 5 (IPO5/KPNA1) and exportin 1 (XPO1) [37].

The three-dimensional (3D) structure of PACS-1 is still unknown. Some attempts have been made using ab-initio modeling, such as the one described for residues located between V_117_ and D_300_, which are predicted as a globular domain [7]. Different unpublished analyses performed using structure prediction systems using hidden Markov models [38,39] roughly coincide in predicting a globular structure for the C-terminal subdomain (residues from R_622_ to L_956_), similar to a regulatory subunit of phosphoinositide 3-kinase. They also predict a structure similar to a C2 domain (calcium/lipid-binding domain) for the segment between amino acids P_98_ and E_261_, which would include the amino acid R_203_ (variant p.R203W). Unfortunately, not all of these data are conclusive. Future knowledge of the structure of PACS-1 would be key to knowing more accurately how the mutations in the protein affect its structure and to developing therapies that are more precise for these patients.

### 3.4. Functions of PACS-1

**Figure 2:**
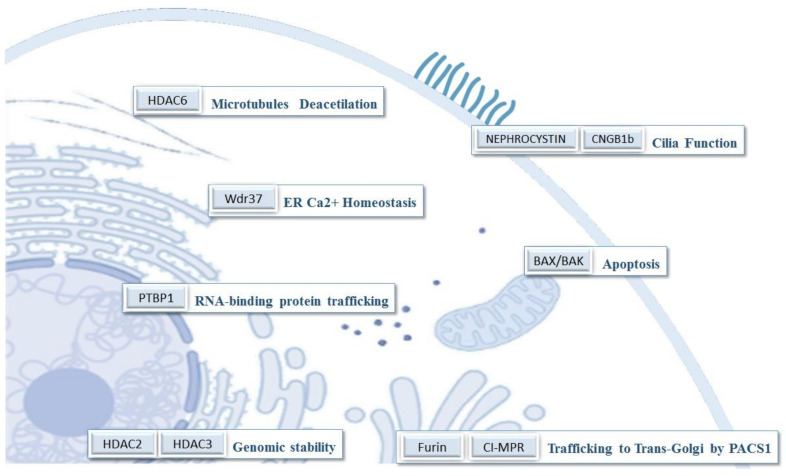
Some of PACS-1’s client proteins and their subcellular location and function.

Nowadays, more than 100 proteins have been described that can putatively bind to PACS-1 [11]. This could explain, in part, the number of different processes where PACS-1 might be involved. Therefore, although the main function of PACS-1 is related to protein trafficking, sometimes it is difficult to discern between the PACS-1 function and the role of some of its client proteins (Figure 2) (Supplementary Table S1).

The first described function of the PACS-1 protein was its role in the regulation of membrane traffic proteins. This specific function is well known and conserved among vertebrates in PACS proteins. The activation mechanism involves the phosphorylation of its S_278_ residue by CK2. The activated PACS-1 protein is able to bind its client proteins, transferring them to their final location. It mediates the trafficking of proteins from the plasma membrane to the early endosomes (SorLA) [40] and from the late endosomes to the trans-Golgi network (TGN) (furin, CI-MPR) [8,41]. Moreover, some proteins related to the function of the primary cilium, such as nephrocystin or CNGB1b, are also trafficking [42,43]. Besides, PACS-1 has been linked to the cytosolic HDAC6, which is involved in the deacetilation of microtubules, in the Golgi integrity and cilium retraction [44,45].

Another function for the PACS family of proteins is connected to the trafficking of proteins related to the apoptosis pathway [10]. PACS2 allows the transferring of Bid to the mitochondria, where it is cleaved to tBid, facilitating the triggering of apoptosis [46]. On the other hand, the downexpression of PACS-1 increases cell survival. The mechanism implies a failure in the BAX/BAK oligomerization, avoiding the mitochondrial outer membrane permeabilization (MOMP) [34]. The increase in cell survival when PACS-1 is downregulated has been linked with a worst prognosis in gastric cancers, and it has been proposed as a biomarker [47].

Moreover, it has been reported that the complex PACS1–Wdr37 facilitates the regulation of calcium flux between the endoplasmic reticulum and the cytosol [11]. The endoplasmic reticulum’s Ca^2+^ release, mediated by the inositol 1,4,5-trisphosphate receptor (IP3R), is regulated by this complex. The deletion of PACS-1 provoked a decrease in the Wdr37 protein, and the deficiency of both caused a reduction in the IP3R expression level. This could explain the overlapping phenotypic characteristics found between the PACS1 and Wdr37 deficiency patients (Table 1).

In the nucleus, PACS-1 binds to PTBP1, a protein involved in the binding and trafficking of RNA [36]. Moreover, it interacts with and could stabilize HDAC2 and HDAC3, contributing to genomic stability [12,35].

## 4. Relationship between PACS-1 Function and the *PACS1*-NDD Patient’s Phenotype

Nowadays, there are a few studies regarding the relationship between PACS-1 function and the *PACS1*-NDD phenotype. It is proposed that the most frequent PACS-1 variant, p.R203W, causes a gain-of-function (GOF) or a dominant negative mechanism [1,48]. However, the truncation of the protein (del ex12-24) with a loss-of-function has been described in two patients with a mild and not characteristic *PACS1*-NDD clinic [6].

The mutated amino acid (R203W) is in the furin cargo-binding domain and in close proximity to the CK2 binding motif (Figure 1), so it is possible that the correct binding and phosphorylation function of CK2 could be compromised [14]. Several experimental approaches have been carried out with a mutant PACS-1 to explore the differences in the binding of PACS-1 to its client proteins. In this sense, some protein–protein interactions are not affected (AP3D1, CLCN7, HDAC2/3) [1,12], whilst others decrease their binding level (TRPV4v2) [1] and others increase it (HDAC6) [45]. On the other hand, experiments in zebrafish embryos where the mutant mRNA of *PACS1* was injected showed a decrease in cranial cartilaginous structures compared to the control. This could be due to the fact that the migration of cranial-neural-crest cells (CNCCs) is related to PACS1 [1].

An experimental model of the *PACS1*-NDD of forebrain organoids has been developed (PACS1^(+/R203W)^), besides the knock-out of the *PACS1* gene (PACS1^(-/-)^). Gene expression pattern experiments showed differences between the PACS1^(+/R203W)^ and the control model, but not between the PACS1^(-/-)^ and the control, supporting the idea that this specific mutation confers a GOF and a toxic effect on the protein. Genes directly related to the autism spectrum disorder (ASD) and to the development of GABAergic synapses are upregulated in the disease model [48]. These results support the previous findings that about 40% of the *PACS1*-NDD patients have been formally diagnosed with autism [49].

These results, in zebrafish and forebrain organoids, could explain, in part, the neurologic impairment development of *PACS1*-NDD patients. Moreover, other proteins related to intracellular trafficking have also been associated with craniofacial diseases [26] and neurodevelopmental disorders [2].

## 5. Therapy for *PACS1* Deficiency Patients

To date, the *PACS1*-NDD syndrome has been symptomatically treated by a multidisciplinary team. The neurodevelopmental alterations recommend an early interventional program, which includes occupational, physical and speech therapy. Anxiety and behavioral problems have been managed with psychotropic drugs. Seizures respond well to the classical epilepticus treatment. It is suggested that early physical therapy treatment for motor dysfunction can improve mobility and decrease the risk of later orthopedic complications. Feeding problems might need nutritional intervention therapy and, in severe cases, a gastrostomy tube [13].

**Figure 3:**
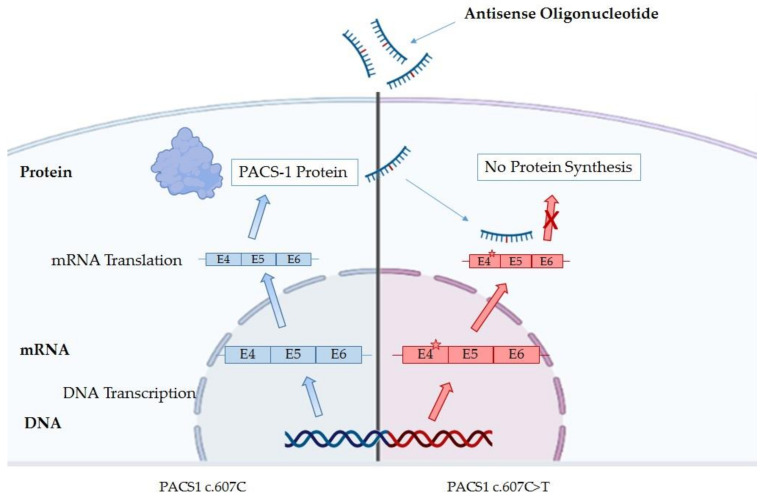
Schematic mechanism of antisense oligonucleotides (ASOs) therapy in order to avoid the translation of the mutant PACS-1 protein.

In recent years, more targeted therapy approaches are being developed for some rare diseases. In this sense, the *PACS1*-NDD is a good candidate [50] because most of the patients carry the same mutation, and a gain-of-function or a dominant negative mechanism has been proposed [1,3,48]. However, the highest expression level of PACS-1 during fetal brain development could limit the effectiveness of the treatment [31,32]. Nowadays, there are four approaches which can be performed; two of them are in more advanced steps due to the use of antisense oligonucleotides (ASOs) (Figure 3) or inhibitors against HDAC6 [45,51]. The other two are subject to advances in the knowledge of the 3D PACS-1 structure [7], as the proteolysis-targeting chimeras (PROTACs) or molecules, which specifically target the mutated protein.

The antisense oligonucleotides (ASOs) can specifically target the mutated mRNA of *PACS1*, avoiding the translation of the pathologic protein [52] (Figure 3). The company IONIS is collaborating with the PACS1 foundation in order to develop an ASO targeting the p.R203W variant [51], and, nowadays, it is an active research field. However, ASOs are incapable of crossing the blood brain barrier (BBB) and require direct central nervous system (CNS) administration, so intrathecal delivery is recommended [53].

Other approaches propose the targeting of some of the client proteins of PACS-1, whose function is altered in these patients. In this sense, the research carried out by Dr. Thomas′s group is interesting [45]. They suggest the specific targeting of HDAC6 through its inhibition. They have delivered a patent application in which, in fibroblasts derived from patients, the stronger binding between the mutated PACS-1 and HDAC6 could be associated with a fragmented Golgi and a different microtubule network compared to the controls. They confirm that the use of general (TSA) or selective (tubacin, ACY-1215 or SW-100) HDAC6 inhibitors rescues the normal cellular phenotype [45].

Finally, two more approaches can be developed that focus on the specific targeting of the mutated PACS-1 protein. The proteolysis-targeting chimeras (PROTACs) bind simultaneously to the target protein and to an E3 ligase, forming a ternary complex, which promotes the ubiquitination of the protein of interest, thereby, inducing proteasomal degradation [54]. On the other hand, the use of molecules that specifically bind to the mutant PACS-1 protein is another way to produce protein degradation. However, the proper development of both technologies needs knowledge of the 3D structure and of the differences between the PACS-1 wild-type and mutated proteins [7].

## 6. Conclusions

In recent years, an extraordinary advance in the study of cellular protein trafficking has been made. PACS-1 is a connector multifunctional protein with a role that trespasses the trafficking between endosomes and the TGN and could be key in cellular homeostasis, interacting with proteins related to apoptosis, genomic stability or calcium flux in the endoplasmic reticulum. Since 2012, when *PACS1*-NDD was first characterized, deep phenotyping analysis has allowed us to establish a potential relationship with other neurodevelopmental disorders, such as the PACS2 or Wdr37 syndromes. However, it is necessary to find a more profound approach that allows us to connect the physiological mechanisms of cellular trafficking and clinical features in order to reclassify and properly understand these genetic disorders.

Exceptionally, most of the *PACS1*-NDD patients share the same mutation, and a gain-of-function or a dominant negative mechanism has been proposed. This makes it a great model for the therapy of rare diseases caused by this mechanism. In this sense, strategies focused on decreasing the toxic effect of the mutated protein by inhibiting its expression with ASOs or its client proteins seem to be promising.

## Figures and Tables

**Table 1:** Clinical characteristics of the *PACS1*-NDD, PACS2, Wdr37, Kabuki and Cornelia de Lange Syndromes.

Clinical Feature	HPO ID *	*PACS1*-NDD	PACS2 Syndrome	Wdr37 Syndrome	Kabuki Syndrome	CdLS
**Neurodevelopmental features**						
Intellectual disability	0001249	Obligate	Very frequent	Obligate	Obligate	Very frequent
Autism spectrum disorder	000729	Occasional	Occasional	Occasional	Occasional	Frequent
Development delay	0012758	Obligate	Very frequent	Obligate	Obligate	Occasional
Speech delay	0000750	Very frequent	Very frequent	Frequent	Occasional	Frequent
Hypotonia	0001252	Frequent	Frequent	Frequent	Frequent	Occasional
Seizures	0001250	Frequent	Very frequent	Very frequent	Occasional	Occasional
**Congenital malformations**						
Dysmorphic facial features						
Full and arched eyebrows	0002553	Frequent	Frequent	Frequent	Very frequent	Very frequent
Hypertelorism	0000316	Frequent	Frequent	Frequent	Occasional	Very rare
Downslanting palpebral fissures	0000494	Frequent	Frequent	Frequent	Very frequent	Very rare
Bulbous nasal tip	0000414	Frequent	Very frequent	Obligate	Frequent	Very frequent
Downturned mouth	0002714	Frequent	Frequent	Frequent	Very rare	Very frequent
Thin upper lip	0000219	Frequent	Very frequent	Very frequent	Occasional	Very frequent
Brain abnormalities						
Hypoplasia or partial agenesis of the cerebellar dermis	0006817	Frequent	Frequent	Obligate	Occasional	Occasional
Ophthalmologic						
Coloboma	0000589	Occasional	Occasional	Very frequent	Occasional	Very rare
Congenital heart anomalies						
Atrial or ventricular septal defects	0001671	Frequent	Occasional	Frequent	Frequent	Frequent
**Others**						
Feeding/GI issues	0011968	Occasional	Occasional	Very frequent	Frequent	Frequent
Skeletal anomalies	0000924	Occasional	Frequent	Frequent	Frequent/ very frequent	Frequent
Cryptorchidism	0000028	Frequent	Frequent	Very frequent	Occasional	frequent

* HPO ID, Human Phenotype Ontology Identifier. Obligate 100%; Very frequent 80–99%; Frequent 30–79%; Occasional 5–29%; Very rare 1–4%; Excluded 0%. Light grey: same disease spectrum.

## Supplementary Materials

The following supporting information can be downloaded at: https://www.mdpi.com/article/10.3390/ijms23179649/s1. References [55,56,57,58,59,60,61,62] are cited in the supplementary materials.
