# Supplementary material for: Molecular Basis of the Schuurs–Hoeijmakers Syndrome: What We Know about the Gene and the PACS-1 Protein and Novel Therapeutic Approaches
Source: Int J Mol Sci. 2022 Aug 25;23(17):9649. doi: 10.3390/ijms23179649 (PMC9456036; doi:10.3390/ijms23179649)
Supplement: Supplementary file 1 [file ijms-23-09649-s001.zip › ijms-1862929-supplementary.pdf]

Supplementary Table S1. PACS1 client proteins, function and location.

Protein	Function	Location	Reference *
Furin	Protease	TGN, cellular membrane	[8]
CI-MPR	Carrier of Man-6-P-containing lysosomal enzymes	Golgi / endosome	[44]
SORLA	Sorting receptor of APP	Secretory pathway	[45]
BACE1	Cleavage of the amyloid precursor protein	Post-endoplasmic reticulum compartments	[55]
VAMP4	Secretory regulation	Golgi / TGN	[56]
CNGB1b	Ion channel	Cilia	[41]
Nephrocystin	Proper cilia function	Cilia base	[40]
PTBP1	pre-mRNA splicing and in the regulation of alternative splicing events	Nucleus / cytosol	[37]
Nef	Endocytosis of MHC-I molecules	Cytosol	[57]
Rev	Regulation of HIV-1 (and other lentiviral) protein expression	Nucleus / cytosol	[58]
BBLF1	Cytoplasmic envelopment	Endosomes and trans-Golgi	[59]
HDAC2/3	Histone deacetylation	Nucleus	[12]
Wdr37	ER Ca^{2+} flux	Cytosol	[11]
HDAC6	Protein deacetylase	Cytosol	[43]
CLCN7	Cloride channel	Lysosome membrane	[1]
TRPV4v2	Ion channel	Endoplasmic reticulum	[1]
TRPP2/PKD2	Ion channel	Cell membrane	[60]
TfR2	Uptake of transferrin-bound iron	Cytosol	[61]
PC6B	Serine endoprotease	Secreted	[62]

* Reference to the PACS1 protein relatioship
